# Neoadjuvant endocrine therapy with sequential palbociclib and chemotherapy based on Ki67 status in stage II-III breast cancer: An open-label, phase II study

**DOI:** 10.1016/j.breast.2025.104672

**Published:** 2025-12-10

**Authors:** Christina Engebrethsen, Synnøve Yndestad, Mari E. Rasmussen, Emiel A.M. Janssen, Bjørnar Gilje, Egil S. Blix, Helge Espelid, Steinar Lundgren, Jürgen Geisler, Laura Minsaas, Reidun Lillestøl, Hildegunn S. Aase, Turid Aas, Per E. Lønning, Hans P. Eikesdal, Stian Knappskog

**Affiliations:** a Department of Clinical Science, University of Bergen, Bergen, Norway; b Cancer Clinic, Haukeland University Hospital, Bergen, Norway; c Department of Pathology, Stavanger University Hospital, Stavanger, Norway; d Department of Chemistry, Bioscience and Environmental Engineering, University of Stavanger, Stavanger, Norway; e Department of Hematology and Oncology, Stavanger University Hospital, Stavanger, Norway; f Immunology Research Group, Institute of Medical Biology, UiT The Arctic University of Norway, Tromsø, Norway; g Department of Oncology, University Hospital of North Norway, Tromsø, Norway; h Department of Surgery, Haugesund Hospital, Haugesund, Norway; i Cancer Clinic, St. Olavs Hospital, Trondheim University Hospital, Trondheim, Norway; j Department of Clinical and Molecular Medicine, Norwegian University of Science and Technology, Trondheim, Norway; k Department of Oncology, Akershus University Hospital, Lørenskog, Norway; l Institute of Clinical Medicine, University of Oslo, Oslo, Norway; m Department of Radiology, Haukeland University Hospital, Bergen, Norway; n Department of Surgery, Haukeland University Hospital, Bergen, Norway; o Institute for Biomedicine and Glycomics, Griffith University, Queensland, Australia

**Keywords:** Neoadjuvant treatment, Hormone receptor-positive breast cancer, Ki67, Endocrine therapy, Aromatase inhibitor, CDK4/6 inhibitor, Biomarkers

## Abstract

**Background:**

While neoadjuvant endocrine therapy (NET), with or without a CDK4/6 inhibitor, is an established treatment option for estrogen receptor-positive breast cancers, optimal patient selection and second-line treatment for non-responders remain uncertain.

**Methods:**

In the open-label, phase 2 PETREMAC trial (NCT02624973), pre- and postmenopausal patients with large T2 (>4 cm) or locally advanced ER/PGR>50 %, HER2-, and *TP53* wild-type breast cancers received NET (tamoxifen + goserelin for premenopausal and letrozole for postmenopausal patients). Palbociclib was added if the Ki67 reduction was ≤50 % after 14 days. Neoadjuvant chemotherapy (NAC) was introduced if NET ± palbociclib failed to reduce Ki67 sufficiently or if there was no objective response on MRI after 24 weeks. Tumor biopsies underwent targeted sequencing of 360 cancer-related genes and subsequent gene expression profiling.

**Results:**

Among 88 patients, the median tumor size was 48 mm (range 16–140 mm). NET alone reduced Ki67 > 50 % in 49/88 (56 %) of evaluable tumors. Adding palbociclib yielded a Ki67 reduction >50 % in 24/34 (71 %) of tumors where neoadjuvant endocrine therapy alone failed to suppress Ki67, providing a Ki67 reduction of >50 % in a total of 72/88 (82 %) of patients. NAC was administered to 34/88 (39 %) due to inadequate Ki67 response or lack of MRI response. Overall, 70 % achieved a pre-surgical objective response. Pathological complete response was seen in 3/84 patients. Postmenopausal status (p = 0.005) and invasive lobular carcinoma (p = 0.02) predicted Ki67-based response to NET.

**Conclusion:**

Sequential NET with palbociclib, limiting NAC to non-responders, is a feasible strategy for ER/PGR>50 %, HER2-, *TP53* wild-type breast cancers.

## Highlights

• Sequential NET with response-guided escalation is feasible in ER+/HER2– BC.
• 82 % of the patients achieved >50 % Ki67 reduction after NET ± palbociclib.
• 70 % of patients achieved an objective pre-surgical response.
• 61 % of patients completed neoadjuvant treatment without need of chemotherapy.

## 1 Background

Neoadjuvant endocrine treatment (NET) is an established treatment option for selected postmenopausal women with large, hormone receptor-positive (HR+)/HER2-negative (HER2-) breast cancers, in particular for the luminal A subtype [1,2]. While randomized studies have indicated a similar treatment efficacy for neoadjuvant chemotherapy (NAC) and NET [[3], [4], [5]], more biomarkers are required for optimal treatment stratification on an individual basis [6], and the optimal second-line treatment for patients having an inadequate response to primary NET remains to be settled. Cyclin-Dependent Kinase 4/6 inhibitors (CDK4/6i) offer a less toxic treatment modality as compared to chemotherapy [7,8], but it remains to be defined which patients may benefit from having a CDK4/6i added in concert with NET, and who will be in need of chemotherapy.

Studies pioneered by Dowsett and co-workers have revealed that a drop in tumor Ki67 expression following 2 weeks of endocrine treatment may predict recurrence-free survival [9] and guide the selection of correct adjuvant treatment [10]. Furthermore, clinical trials have demonstrated a significant drop in Ki67 by the addition of a CDK4/6i to patients failing on NET alone [11,12].

PETREMAC was an open-label, phase 2, multi-center trial allocating patients with large (>4 cm diameter±node metastases) breast tumors to different treatment arms based on estrogen receptor (ER)/progesterone receptor (PGR) and HER2 expression and *TP53* tumor mutation status [13]. Here, patients with ER+/PGR+ (>50 %), HER2-, and *TP53* wild-type breast cancers received primary NET with the addition of a CDK4/6i (Palbociclib) in case of insufficient Ki67 suppression following 2 weeks on NET monotherapy. Pre-surgical chemotherapy was administered only to those who did not achieve adequate Ki67 suppression on NET±CDK4/6i or revealed a lack of clinical response.

The aim was to evaluate the reduction of Ki67 after 2 and 5 weeks of treatment, focusing on using this early marker to guide therapy escalation and prospectively assess the predictive and prognostic value of molecular alterations.

## 2 Patients and methods

### 2.1 Study design and patients

The PETREMAC (PErsonalized TREatment of high-risk MAmmary Cancer) trial (Clinicaltrials.gov #NCT02624973) was an open-label, multicenter phase 2 clinical trial, where patients with primary breast cancers >4 cm largest diameter or N+, M0, were allocated to eight different treatment arms, based on hormone receptor status (ER, PGR), HER2 expression and *TP53* mutation analyses (Appendix A: Fig. S1). The recruitment period lasted from April 21st, 2016, until August 28th, 2018. Results from patients with triple-negative tumors (arms G and H) [13], and the distribution of mutations related to Homologous Recombination Deficiency (HRD) across all arms, are previously published [14]. In PETREMAC, patients with ER/PGR >50 %, HER2-, and *TP53* wild-type tumors were assigned to arm A (Appendix A: Fig. S1). Premenopausal women received p.o tamoxifen 20 mg daily and s.c goserelin 3.6 mg every 4 weeks, while postmenopausal patients received p.o letrozole 2.5 mg daily, both for 24 weeks.

Due to the multi-arm design, power estimates for each treatment arm were not conducted. The protocol aimed at enrolling at least 200 patients. Evaluation of response rates toward individual treatment regimens, and potential identification of predictive biomarkers, should be basis for independent confirmatory studies.

The CDK4/6i palbociclib was added if NET failed to reduce Ki67 by >50 % during the first 14 days of treatment (Appendix A: Fig. S1). Palbociclib was administered at 125 mg p.o. once daily for three out of four weeks (one cycle = 4 weeks). If the Ki67 reduction remained ≤50 % after 3 weeks, patients were switched to chemotherapy. In case of a Ki67 reduction >50 %, patients continued treatment with NET ± palbociclib for up to 24 weeks or until disease progression by MRI and/or clinical assessment. Patients not obtaining an objective response (CR/PR) on MRI after 24 weeks received subsequent NAC. The NAC regimen included docetaxel 80 mg/m2 i.v. every 2 weeks or paclitaxel 80 mg/m2 i.v. weekly. If the response to taxane treatment was insufficient, there was crossover to epirubicin monotherapy 90 mg/m2 i.v. every 2 weeks. Sequential rather than concurrent administration of taxane and anthracycline was chosen, as large meta-analyses show that sequential regimens are equally or superiorly efficacious with lower toxicity [15,16]. The sequence of taxane and anthracycline does not affect pCR or survival outcomes [17], and the taxane-first approach was therefore adopted for practical and tolerability reasons, in line with the study protocol (Appendix B). All patients were included with surgical intent; use of palbociclib and/or chemotherapy did not alter surgical planning. For details, see Appendix B: Study protocol.

The decision to use a relative drop in Ki67 over an absolute cut-off (like 10 %) was based on previous findings indicating that a significant reduction in Ki67 after 2 weeks of endocrine therapy predicts both response and relapse-free survival [9]. By measuring a percentage decrease rather than an absolute threshold, the study ensures that patients with initially high Ki67 levels, who may still have substantial residual Ki67 expression after treatment, are not falsely classified as non-responders.

Patients underwent caliper measurements every four weeks to assess clinical response to avoid unnecessary delay in case of progressive disease. Breast MRI was performed during pretreatment screening and after eight weeks of treatment. At 24 weeks, MRI was repeated, and operable patients were referred for surgery. Response evaluation followed the RECIST guidelines [18], except from progressive disease (PD), where the UICC criteria [19] were used. UICC defines PD as an increase >25 % in the product of the largest tumor diameter and its perpendicular diameter, contrasting the RECIST definition of >20 % increase in one diameter. This conservative approach aimed to prevent unnecessary growth of large tumors. For evaluations with both caliper and MRI, OR results were based on MRI assessment. Where MRI exams were absent, caliper measurements were used for OR assessment.

Tru-cut biopsies or vacuum biopsies for biomarker analysis were collected before and after each treatment introduced as per the study protocol (Appendix A: Fig. S1 and Appendix B: Study Protocol).

### 2.2 Histology and immunohistochemistry (IHC)

Local pathologists analysed formalin-fixed paraffin-embedded (FFPE) biopsies at each trial site. Hematoxylin and eosin staining (4 μm sections) classified samples as infiltrating lobular carcinoma (ILC) or infiltrating carcinoma of no specific type (IC NST). ER, PGR, and HER2 analyses were conducted following institutional guidelines at each trial site.

Ki67 and E-cadherin immunostaining was performed on Tru-cut/Vacuum biopsies (FFPE), as outlined in Appendix A Supplementary methods, at one centralized laboratory (Stavanger University Hospital).

### 2.3 DNA and RNA analyses

Targeted sequencing of a 360-gene cancer panel, RNA sequencing, and gene expression-based subgrouping of pretreatment tumor biopsies was performed as previously described [13,14,20], and outlined in Appendix A: Supplementary Methods.

### 2.4 Statistical analyses

Fisher's Exact test was used for 2x2 and 2x3 group comparison. A binary logistic regression model was utilised for multivariate analysis. All P-values are two-tailed, with no corrections for multiple testing. Analyses were conducted using SPSS 29.0.2.0 or R version 4.1.3. The test for Fisher's Exact 2x3 was provided by cog-genomics [21].

## 3 Results

### 3.1 Patients and tumor characteristics

Out of 201 patients included in the PETREMAC trial (Appendix A: Fig. S1), 88 patients (44 %) were allocated to arm A; see Table 1 for baseline characteristics. At inclusion, patients in arm A had a median tumor diameter of 51 mm (range 20–110 mm) measured clinically (calipers) and 48 mm (range 16–140 mm) measured by MRI. One or both pretreatment measurements were >40 mm for all patients, except for one patient who was included with a primary tumor <40 mm, having an axillary node >20 mm.

**Table 1:** Baseline characteristics for patients in the PETREMAC trial, arm A.

Parameter	Overall n = 88 (%)
**Accrual period, mo.yr**	05.2016–08.2018
**Median age (range)**	52 (33–78)
**Menopausal status**	
**Premenopausal**	42 (48 %)
**Postmenopausal**	46 (52 %)
**Caliper tumor diameter, mm**	
**Range**	20–110^a^
**Median**	51
**Mean**	54
**MRI tumor diameter, mm**	
**Range**	16–140^b^
**Median**	48
**Mean**	53
**T stage (clinical, caliper)**	
**T1**	1 (1 %)^c^
**T2**	39 (44 %)
**T3**	45 (51 %)
**T4**	3 (3 %)
**N stage (clinical, caliper)**	
**N0**	68 (77 %)
**N1**	14 (16 %)
**N2**	5 (6 %)
**N3**	1 (1 %)
**M stage (radiological)**	
**M0**	86 (98 %)
**M1**^d^	2 (2 %)
**Histology**	
**Infiltrating carcinoma NST**	65/62^e^ (74/70 %)
**Infiltrating lobular carcinoma**	23/26^e^ (26/30 %)
**Tumor grade**^f^	
**1**	3 (3 %)
**2**	17 (19 %)
**3**	3 (3 %)
**NA**^g^	65 (74 %)
**Receptor status**^h^	
**ER**^i^	
**Positive ≥50 %**	88 (100 %)
**PGR**^j^	
**Negative**	9 (10 %)
**Positive <50 %**	19 (22 %)
**Positive ≥50 %**	60 (68 %)
**HER2**^k^	
**Negative**	88 (100 %)
**Ki67**^l^	
**<10 %**	12 (14 %)
**10–50 %**	67 (76 %)
**>50 %**	9 (10 %)
**PAM50 subtype**	
**Luminal A**	45 (51 %)
**Luminal B**	32 (36 %)
**Normal-like**	7 (8 %)
**HER2-enriched**	1 (1 %)
**Basal-like**	0
**NA**^g^	3 (3 %)

a If clinical value< 4 cm, MRI value is > 4 cm except one, where node was ≥2 cm.

b If MRI value< 4 cm, clinical value is > 4 cm except one, where node was ≥2 cm.

c MRI measurement > 4 cm.

d Late diagnosis of M1, patient included as "intention-to-treat”.

e Before/after centralized review.

f Grade 1–2 classified as grade 2, grade 2–3 classified as grade 3.

g No tissue for analysis.

h ER, PGR, HER2 status evaluated at local lab; central lab results were used if local lab analyses failed.

i ER negative: ER <1 %.

j PGR cut-off value < 10 %.

k HER2 negative: HercepTest IHC 0–1+ or IHC 2+ and no HER2 amplification by in situ hybridization.

l Classified at central lab.

In pretreatment biopsies, the median Ki67 value was 23.5 % (range: 1–75 %). Twelve tumors had a Ki67 value of 0–10 %, 37 tumors 11–25 %, 30 tumors 26–50 %, and 9 tumors had a Ki67 > 50 % before commencing NET.

**Fig. 1:**
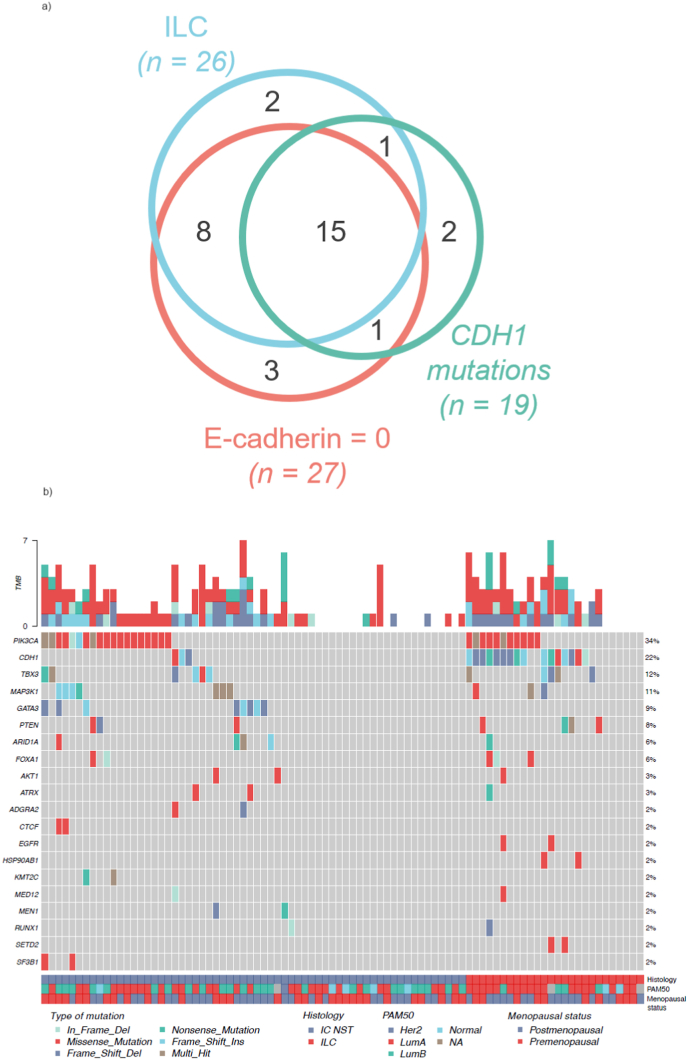
A: Venn diagram of invasive lobular carcinoma, e-cadherin immunohistochemistry staining score = 0, and CDH1 mutations. n = 86 (of 88, n = 2: insufficient material for analysis). ILC, invasive lobular carcinoma **B:** Oncoplot of stage II-III ER/PGR+ >50 %/HER2-and *TP53* wt breast cancers in the PETREMAC trial. Somatic mutations in pretreatment samples are presented. Genes are restricted to those affected by mutations in two or more patients. Out of a total of 88 tumors analysed, 19 of the 88 tumors had no mutations in the gene panel tested. IC NST, invasive carcinoma of no special type; ILC, invasive lobular carcinoma; LumA, luminal A; LumB, luminal B; NA, not analysed; TMB, tumour mutational burden.

Local pathologists at the seven trial centers classified 23 out of 88 tumors as ILC. Among the 65 tumors initially classified as IC NST tumors, four harbored *CDH1* mutations and revealed no E-cadherin staining; three of these were re-categorized as ILC by local pathologists at subsequent surgical specimens, whereas one remained classified as IC NST. Thus, 26 tumors were classified as ILC including pretreatment biopsies and surgical specimens after neoadjuvant treatment; this number was used for further analyses (Fig. 1A). In a centralized, post hoc analysis, 27 out of 86 tumors available for IHC had no E-cadherin staining, including 23 out of 26 ILCs and 4 out of 60 IC NSTs. 16 of the 27 tumors with no E-cadherin staining harbored *CDH1* mutations, while 3 *CDH1* mutated tumors revealed E-cadherin staining (Fig. 1A). 10/16 (63 %) *CDH1* mutations among the ILC patients were frameshift mutations and 2/3 (67 %) *CDH1* mutations among the IC NST patients were frameshift mutations (Fig. 1B).

### 3.2 Ki67

**Fig. 2:**
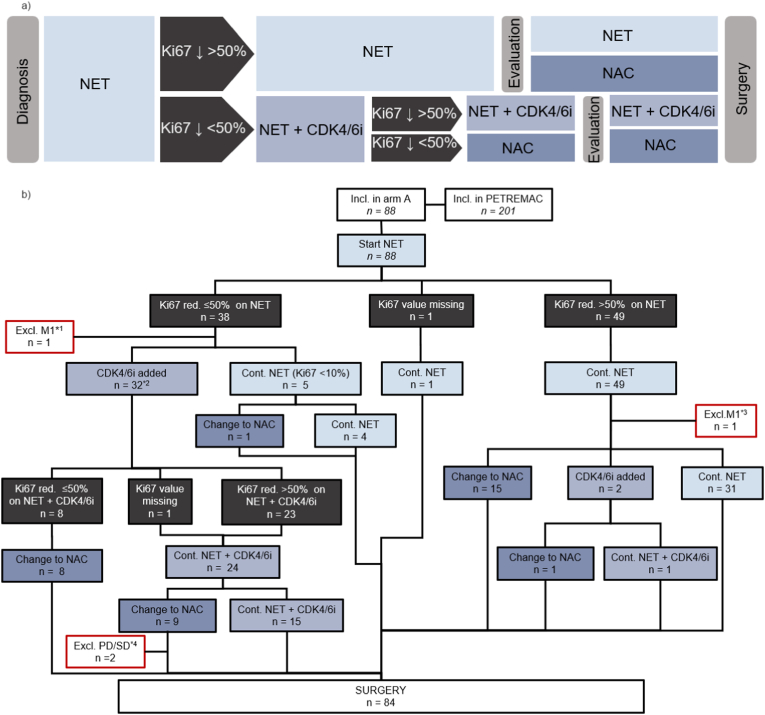
A: Schematic overview of planned treatment flow for the PETREMAC trial, arm A B: CONSORT diagram for the PETREMAC trial, arm A. Treatment was directed by requirements for Ki67 response (>50 % reduction from baseline) and subsequent tumor regression (objective response; OR) as follows: Patients received initial neoadjuvant endocrine treatment (NET); letrozole for postmenopausal patients and tamoxifen + goserelin for premenopausal patients. NET was continued if Ki67 decreased >50 %, if not a CDK4/6 inhibitor (CDK4/6i), palbociclib, was added. If Ki67 decreased <50 % after 3 weeks of combined NET + CDK4/6i, patients were switched to neoadjuvant chemotherapy (NAC; docetaxel). In addition, NAC was implemented if patients did not achieve an objective response (OR) to NET±CDK4/6i. NET, neoadjuvant endocrine treatment; CDK4/6i, CDK4/6 inhibitor; NAC, neoadjuvant chemotherapy; ORR, objective response rate (MRI, caliper measurements were used if MRI was not available); Cont., continued therapy; Excl., excluded; Incl., included; Ki67 < 10 %: Ki67 < 10 % on pretreatment values; n, number of patients; M1, distant metastasis; PD, progressive disease; red., reduction. ∗^1^ Distant metastases (M1 disease) evident on delayed results of baseline radiology, after the patient had commenced on NET ∗^2^ n = 3 had pretreatment Ki67 < 10 % ∗^3^ Distant metastases (M1 disease) suspected at baseline radiology; confirmed by biopsy after the patient had commenced NET ∗^4^ Both patients had pretreatment characteristics as follows: Age >55, tumor size (MRI) ≥ 70 mm, infiltrating carcinoma (non lobular), luminal B, Ki67 > 20 %, and the patient with PD: progesterone receptor 10 % positive. (For interpretation of the references to colour in this figure legend, the reader is referred to the Web version of this article.)

Of the 88 patients who commenced on NET, NET alone resulted in a Ki67 reduction of >50 % in 49/88 (56 %) (Fig. 2). Ki67 analysis at week 2 was unavailable for one patient due to insufficient biomaterial.

Among the 49 initial responders, two patients later received palbociclib due to insufficient clinical response as judged by the treating physician (this was not according to protocol), 31 completed presurgical treatment with NET only, while 16/49 received subsequent chemotherapy due to lack of an objective tumor response. Among the 38 patients without an adequate Ki67 response to NET, 32 received palbociclib; of these, 17 progressed to chemotherapy due to no Ki67 reduction after 3 weeks or no clinical response, as described in the methods section. In total, 34 patients started palbociclib, including three with pretreatment Ki67 < 10 %. NET + palbociclib resulted in a Ki67 reduction >50 % in 24/34 patients (71 %) (analysis after palbociclib was missing for two; Fig. 2B). Therefore, in the intention-to-treat population, 72 out of 88 (82 %) patients receiving NET, with or without palbociclib, achieved a Ki67 reduction >50 % (Fig. 2B). Among these patients, 25/72 did not obtain a sufficient clinical response and were switched to NAC. In total, 34/88 (39 %) patients were switched to NAC due to a Ki67 reduction of less than 50 % or the absence of an OR on NET with or without palbociclib.

Among the 12 patients with pretreatment Ki67 levels below 10 % (Ki67-low), eight did not experience a >50 % reduction in Ki67. Of these, five continued NET treatment (a protocol violation), while three received additional palbociclib as per protocol. Out of these eight patients, three patients required chemotherapy due to SD on MRI at week 24. Presurgical response was: 1 CR, 2 PR, and 5 SD (the latter includes the three patients escalated to NAC, one without MRI report at evaluation and one labelled PR by the investigator but RECIST-classified as SD). Conversely, the four patients with Ki67 < 10 % who did achieve a >50 % reduction in Ki67 on NET remained on NET alone until surgery, with three showing PR and one SD (the latter being a lobular tumor not adequately visualised on MRI, necessitating caliper measurement for response assessment).

Among the 76 patients with a pretreatment Ki67 ≥ 10 %, 45 (59 %) experienced a Ki67 reduction of more than 50 % on NET.

46/88 (53 %) patients were postmenopausal and received letrozole, while 42/88 (47 %) were premenopausal and treated with tamoxifen + goserelin (see Table 1). Among postmenopausal women, 32/45 (71 %) achieved a Ki67 reduction >50 % on letrozole. Conversely, endocrine treatment was less effective in premenopausal patients, with 17/42 (40 %) reaching the same Ki67 reduction on tamoxifen + goserelin (p = 0.005; see Table 2). With NET + palbociclib, a Ki67 reduction >50 % was observed in 7/11 (64 %) postmenopausal and in 17/23 (74 %) premenopausal patients. Overall, in the intention-to-treat population, 39/46 (85 %) of postmenopausal patients and 33/42 (79 %) of premenopausal patients experienced a Ki67 response with NET ± palbociclib (p = 0.3).

**Table 2:** Response to neoadjuvant treatment in arm A of the PETREMAC trial, split by different subgroups.

**a.**		**Ki67 drop on NET**^a^					
	**Total**	**<50 %**	**>50 %**	***p value***	**no OR**^b^	**OR**^b^	***p value***
**Histology**							
IC NST	62 (70 %)	32	29	0.02	17	45	0.6
ILC	26 (30 %)	6	20		9	17	
**PAM50 subtype**^c^							
Luminal A	47 (53 %)	17	30	0.1	14	33	0.8
Non luminal A	38 (43 %)	20	17		10	28	
**Total**	**88**	**38**	**49**		**26**	**62**	

**b.**		**Ki67 drop on NET**^a^					
	**Total**	**<50 %**	**>50 %**	***p value***	**no OR**^b^	**OR**^b^	***p value***
**Menopausal status**							
Postmenopausal	46 (52 %)	13	32	0.005	18	28	0.06
Premenopausal	42 (48 %)	25	17		8	34	
**IC NST**							
Postmenopausal	30 (34 %)	13	16	0.3	12	18	0.05
Premenopausal	32 (36 %)	19	13		5	27	
**ILC**							
Postmenopausal	16 (18 %)	0	16	<0.001	6	10	1.000
Premenopausal	10 (11 %)	6	4		3	7	
**Total**	**88**	**38**	**49**		**26**	**62**	

Patients with stage II-III breast cancers, ER/PGR+>50 %, HER2-, *TP53* wt are included in arm A, the response was recorded as Ki67 response after 14 days of neoadjuvant endocrine treatment and objective response (OR) after completion of neoadjuvant treatment. **a.** ILC vs IC NST, PAM 50 subtype; Luminal A vs non-luminal A breast cancers. **b.** Pre-vs postmenopausal patients; all histological subtypes combined, or within the IC NST and ILC subgroups.

IC NST, invasive carcinoma of no special type; ILC, invasive lobular carcinoma; NA, No tissue for analysis; OR, objective response.

a One patient was excluded from Ki67 analysis due to lack of adequate biomaterial.

b OR assessments were based on MRI evaluation; if not available, caliper measurements were used.

c Three patients had no material for risk-score analysis (n = 85).

In terms of breast cancer histology, 20/26 (77 %) of patients with lobular carcinomas (ILCs) demonstrated a reduction in Ki67 > 50 % during NET monotherapy, compared to 29/61 (48 %) of IC NSTs (p = 0.02, Table 2). This mainly stems from different response rates among subgroups of patients with ILC; among postmenopausal women (receiving letrozole), all had a Ki67 reduction greater than 50 %. In contrast, only 4 out of 10 ILCs in premenopausal women demonstrated an adequate Ki67 response to tamoxifen + goserelin (p < 0.001; Table 2). It is notable that in patients with IC NST tumors, treatment with letrozole in postmenopausal women resulted in a slightly better Ki67 response, with 16 out of 29 (55 %) compared to 13 out of 32 (41 %) for tamoxifen + goserelin in premenopausal women (p = 0.3).

### 3.3 Clinical response and surgery

**Fig. 3:**
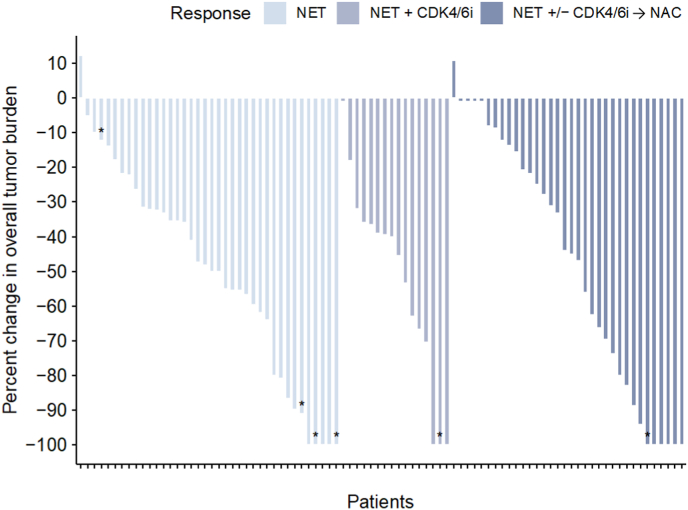
Response to neoadjuvant treatment for HR+/HER2-and *TP53* wild-type breast cancers in the PETREMAC trial. A dotted red line indicates partial response (PR). The response was assessed just prior to surgery, MRI was the primary evaluation method, but for 6 patients clinical evaluation (caliper) was used, and ∗ mark these bars. For 3 patients, an MRI was not performed: one declined due to claustrophobia, another left the trial after a few weeks (incorrectly included M1), and no reason was provided for the third. The clinical response for this patient was PR by calliper measurement (Ki67 < 10 pretreatment and received NET alone). For the remaining three cases, caliper measurements versus MRI did not change from no OR to OR in two cases. In the last case, MRI was regarded as SD by definition but was described as a tumour bed visible (same size as the original tumour), with benign/fibrous signal. Therefore, based on the PI's judgement, this was interpreted as a response, and the patient proceeded to surgery. Responses are categorized by treatment: NET, neoadjuvant endocrine therapy; CDK4/6i, CDK4/6 inhibitor (palbociclib); NAC, neoadjuvant chemotherapy (docetaxel). (For interpretation of the references to colour in this figure legend, the reader is referred to the Web version of this article.)

Considering clinical responses, 62/88 (70 %) patients had an OR, 43 out of 54 (80 %) among those receiving NET±palbociclib, and 19 out of 34 (56 %) among those in need of NAC; Fig. 3 and Table 3. Among the 11 patients who only received NET±CDK4/6i but did not achieve an OR on MRI (see Table 3), 10 out of 11 showed OR when measured by caliper. The one remaining patient was excluded after only a few weeks due to incorrect inclusion, although initially receiving treatment as per the study protocol (Fig. 2B).

**Table 3:** Objective response (OR; partial or complete response) for patients after completing neoadjuvant treatment.

	Total	no OR^a^	OR^a^
**NET**	38	9	29
**NET + CDK4/6i**	16	2	14
**NAC**	34	15	19
**Total**	**88**	**26**	**62**

Patients are grouped by treatments needed to facilitate OR and subsequent surgery.

CDK4/6i, CDK4/6 inhibitor; NAC, neoadjuvant chemotherapy; NET, neoadjuvant endocrine therapy; OR, objective response.

a OR assessments were based on MRI evaluation; if not available, caliper measurements were used.

The complete neoadjuvant treatment regimen, including endocrine therapy with or without palbociclib and chemotherapy, was equally effective in premenopausal and postmenopausal women in terms of tumor regression and overall response (Table 2).

After completing neoadjuvant treatment, 33/84 (39 %) patients had breast-conserving surgery (Table 4), and four patients did not proceed to surgery: one due to PD, one due to SD, and two following the delayed diagnosis of metastatic disease (Fig. 2). pCR occurred in 3/84 (4 %) patients, they had IC NST and received NAC after NET±palbociclib. At 5 years follow-up 7/84 patients have experienced relapse, a fraction as expected for this patient-group (Appendix A: Fig. S2).

**Table 4:** Type of surgery and pathological complete response rates; split by treatment regimes.

	Total	NET±CDK4/6i	NAC
**Breast-conserving surgery**	33 (39 %)	20 (38 %)	13 (41 %)
**Mastectomy**	44 (52 %)	26 (50 %)	18 (56 %)
**Bilateral surgery**	7 (8 %)	6 (12 %)	1 (3 %)
**pCR**	3 (4 %)	0 (0 %)	3 (9 %)
**Total**	**84**	**52**	**32**

CDK4/6i, CDK4/6 inhibitor (palbociclib); NAC, neoadjuvant chemotherapy; NET, neoadjuvant endocrine therapy; pCR, pathological complete response.

### 3.4 Genetic aberrations, gene expression profiles, and correlation to treatment response

Somatic tumor mutations are depicted in Fig. 1B. Due to strong co-variance between lobular status and *CDH1* mutations (p = <0.001; Fig. 1A), the two parameters could not be combined in a single multivariate model. Including either lobular histology or *CDH1* mutation status in multivariate models with menopausal status, ILC remained significantly associated with initial Ki67 response to NET (p = 0.03) while significance was lost for *CDH1* mutation status (p = 0.07). The association between postmenopausal status and response remained significant in both models (p = 0.009 and p = 0.011, respectively). Apart from this, no single mutation correlated significantly with Ki67 response or OR to any treatment regime, nor the final OR evaluated prior to surgery.

In PAM50 subtyping 45 (51 %) of the tumors were classified as luminal A, 32 (36 %) as luminal B, 7 (8 %) as normal-like, and 1 (1 %) as HER2-enriched (Table 1). The correlation between gene expression signatures and treatment flow is outlined in Table 5. Notably, while the percentage of patients in need of chemotherapy was similar among those with tumors classified as Luminal A (19/47; 40 %) compared to those classified as “non-Luminal A” (14/38; 37 %), the PAM50 ROR-C score [22] reported that patients in the intermediate/high-risk group were more likely to require chemotherapy (21/36; 58 % versus 12/49; 25 %; p = 0.007; Table 5). It is worth noting that our patient cohort generally had larger tumors than those for which Prosigna was validated [23]. No significant difference in the need for chemotherapy was recorded between patients assigned to different groups based on the Oncotype DX, MammaPrint, or EndoPredict expression signatures.

**Table 5:** Treatment implemented in arm A of the PETREMAC trial in different subgroups and risk scores.

	NET	NET + CDK4/6i	→ NAC	*p value*^b^
**Menopausal status**				
Postmenopausal	27	4	15	**0.004**
Premenopausal	11	12	19	
**Histology**				
IC NST	23	11	28	0.1
ILC	15	5	6	
**PAM50 subtype**				
Luminal A	20	8	19	0.9
Non luminal A	16	8	14	
NA	2	0	1	
**Total**	**38**	**16**	**34**	
**PAM50 ROR score**^a^				
Low	26	11	12	**0.007**
Intermediate/high	10	5	21	
**Oncotype DX RS score**^a^				
Low	16	8	10	0.3
Intermediate/high	20	8	23	
**MammaPrint**^a^				
Low	15	5	8	0.3
High	21	11	25	
**EndoPredict**^a^				
Low	15	3	7	0.2
High	21	13	26	
**Total**^a^	**36**	**16**	**33**	

CDK4/6i, CDK4/6 inhibitor; IC NST, invasive carcinoma of no special type; ILC, invasive lobular carcinoma; NA, No tissue for analysis; NAC, neoadjuvant chemotherapy; NET, neoadjuvant endocrine therapy.

a Three patients had no material for risk score analysis (n = 85).

b P-value; Fisher exact test (2-sided).

### 3.5 Safety

Adverse events (AE) are outlined in Appendix A: Table S1 and were according to expectations for the therapies used.

## 4 Discussion

Previous randomized studies have shown that NET can provide clinical benefits and response rates similar to chemotherapy in postmenopausal breast cancer patients with HR+, T2 or larger tumors [[3], [4], [5]]. The main advantage of NET over chemotherapy lies in its reduced short- and long-term toxicity, but beyond positive ER receptor status, criteria for selecting optimal candidates for NET remain limited.

By implementing Ki67 as an evaluation method, as in WGS-ADAPT [24], treatment decisions can be made earlier by assessing Ki67 levels after two weeks, unlike clinical responses that may take months. Studies such as POETIC and IMPACT show that Ki67 suppression after two weeks of therapy predicts improved recurrence-free survival. [9,10,25,26], making it a valuable predictive marker for clinical outcome. This is further supported by a recent systematic review and meta-analysis demonstrating that a high Ki67 after neoadjuvant endocrine therapy is associated with worse survival outcomes in ER-positive/HER2-negative early breast cancer [27]. Since CDK4/6i works by slowing down the cell cycle [12], it is likely that Ki67 suppression may be a valid marker for response to such therapy as well. Yet, the value of adding CDK4/6i for patients with inadequate Ki67 suppression on NET alone remains unsettled.

Adding CDK4/6i in concert with endocrine therapy offers promising benefits both in metastatic [[28], [29], [30], [31], [32]] and adjuvant [33,34] settings, although recent data from the neoadjuvant setting are conflicting [12]. Evidence linking cyclin expression to resistance to CDK4/6i has been discrepant [35,36], and they have not been validated for clinical use. The results from the randomized PALLET study revealed no significant improvement in clinical response rate by adding palbociclib to letrozole in the neoadjuvant setting [12], in concert with the potential side effects and costs related to CDK4/6i therapy [29,37,38] this argues for exploring the benefits of such therapy in selected subgroups based on outcome to NET therapy. We assessed the benefit of adding CDK4/6i for patients with sub-optimal responses using NET alone, as evaluated by short-term Ki67 suppression. This resulted in a sufficient Ki67 response in 71 % of patients after 3 weeks on combined NET±CDK4/6i. The fact that only 4 out of 12 patients with tumors exhibiting pretreatment Ki67 below 10 % obtained >50 % drop in Ki67 likely reflects technical limitations in detecting further suppression within this low range. Only three of the Ki67 low patients needed chemotherapy based on pre-surgery MRI evaluations, aligning with POETIC trial [10] findings that this group does well on NET±CDK4/6i. Notably, while palbociclib was used in the present study, other CDK4/6 inhibitors (abemaciclib and ribociclib) are currently implemented in clinical practice as well, but we believe the PETREMAC concept and results may be extrapolated across CDK4/6 inhibitors.

ArmA of the PETREMAC-trial was restricted to ER/PGR>50 %, HER2-, *TP53* wild-type tumors. While the majority of ER/PGR>50 %, HER2-are *TP53* wild-type, across data sets, some are also mutated (in the PETREMAC trial; n = 88 wild-type versus n = 17 mutated). This potential difference should be taken into account when comparing our results to data sets unselected for *TP53* status.

In the present study, we conducted molecular analysis using a 360-gene panel and evaluated tumor phenotypes to identify additional predictive markers. Except for an improved Ki67 suppression in tumors with *CDH1* mutations, which strongly co-varies with lobular histopathology, no genetic markers predicted treatment response. Further studies are warranted to define the precise role of *CDH1* mutations and/or lobular histopathology in this respect. While E-cadherin plays a complex role in cancer development, not only related to epithelial-to-mesenchymal transition and loss of cell adhesion but also to intracellular processes like regulation beta-catenin function [[39], [40], [41], [42]], it is currently unclear whether or how these functions might influence endocrine sensitivity. The improved Ki67 response to aromatase inhibitors in postmenopausal women compared to tamoxifen + ovarian function suppression (OFS) in premenopausal women with lobular histology aligns with findings from the BIG 1–98 Trial on tamoxifen monotherapy vs aromatase inhibitors for postmenopausal ILC patients [43]. Our study and results from trials in the adjuvant [44,45] and neoadjuvant [46] settings suggest that postmenopausal ILC patients may be prime candidates for investigating chemotherapy omission. While the fraction of ILC in the present study was somewhat higher than expected, we believe this has occurred by chance.

Given the widespread use of gene expression signatures, it may be that a prediction of which patients could benefit from CDK4/6 inhibitors and which could omit chemotherapy, would be possible in larger aggregated data. However, such signatures did not have any predictive value in our study. We believe additional predictive biomarkers are essential to better determine which patients would benefit from the addition of CDK4/6i to NET treatment versus those who require chemotherapy.

Except for the PAM50 ROR score, none of the gene expression signatures implemented for adjuvant therapy predicted outcomes in our material.

## 5 Conclusions

This study demonstrates that a sequential approach, starting with NET and using Ki67 levels and clinical response to guide the addition of CDK4/6i and chemotherapy, is both feasible and effective for ER/PGR>50 %, HER2-and *TP53* wild-type breast cancers. We believe this to be a feasible approach also for further exploration of molecular markers predicting Ki67 suppression, as well as Ki67 suppression after sequentially adding CDK4/6i in patients not responding to NET.

## CRediT authorship contribution statement

**Christina Engebrethsen:** Writing – review & editing, Writing – original draft, Visualization, Methodology, Formal analysis, Data curation. **Synnøve Yndestad:** Writing – review & editing, Methodology, Formal analysis. **Mari E. Rasmussen:** Data curation, Formal analysis, Visualization. **Emiel A.M. Janssen:** Writing – review & editing, Methodology, Data curation. **Bjørnar Gilje:** Writing – review & editing, Investigation. **Egil S. Blix:** Writing – review & editing, Investigation. **Helge Espelid:** Writing – review & editing, Investigation. **Steinar Lundgren:** Writing – review & editing, Investigation. **Jürgen Geisler:** Writing – review & editing, Investigation. **Laura Minsaas:** Writing – review & editing, Methodology. **Reidun Lillestøl:** Writing – review & editing, Methodology, Data curation. **Hildegunn S. Aase:** Writing – review & editing, Investigation. **Turid Aas:** Writing – review & editing, Investigation. **Per E. Lønning:** Writing – review & editing, Writing – original draft, Supervision, Project administration, Investigation, Funding acquisition, Conceptualization. **Hans P. Eikesdal:** Writing – review & editing, Writing – original draft, Supervision, Project administration, Investigation, Funding acquisition, Data curation, Conceptualization. **Stian Knappskog:** Writing – review & editing, Writing – original draft, Supervision, Project administration, Funding acquisition, Formal analysis, Data curation, Conceptualization.

## Previous presentation

An abstract of the current manuscript was presented as a poster discussion at the annual ESMO Conference in Barcelona on October 1, 2019.

## Ethics approval and consent to participate

The PETREMAC trial was registered at Clinicaltrials.gov (NCT02624973) and with EudraCT (#2015-002816-34) and approved by The Norwegian Drug Agency (#2015/8463) and the Regional Ethical Committee of the Western Health Region in Norway (#2015/1493). The study was conducted under provisions of the Declaration of Helsinki, the study protocol, good clinical practice guidelines, and all local regulations. An informed consent form was signed by all patients at inclusion. See Appendix B: Study Protocol for details regarding clinical trial design and conduct. Legal entity responsible for the study: Haukeland University Hospital, Bergen, Norway.

## Consent for publication

Not applicable.

## Availability of data and materials

The datasets used and analysed from the PETREMAC trial are available from the corresponding author upon reasonable request.

## Declaration of generative AI and AI-assisted technologies in the writing process

When finalising the manuscript, Christina Engebrethsen used Grammarly from Grammarly Inc. to improve the readability and language of the manuscript. After using this tool, the author(s) reviewed and edited the content as needed and take(s) full responsibility for the content of the publication.

## Funding

This work was funded by unrestricted grants from The K.G. Jebsen Foundation [SKGJ-MED-020 to HPE, SK, PEL], The Norwegian Health Region West [912008 to PEL], The Norwegian Research Council [273354 to PEL] and The Norwegian Cancer Society [190281 to SK, 190275 to PEL and 223204 to SK]. Research funding was provided by 10.13039/501100007872Grieg Foundation (to HPE and 10.13039/100011328TA), The Norwegian 10.13039/100018696Health Region West [912252; Clinical researcher fellowship to HPE], and 10.13039/100010905Illumina [9529854, to HPE, SK, PEL]. 10.13039/100004319Pfizer [WI206347] provided trial funding and study medication to HPE, SK, and PEL. The funders had no role in the study design, data collection, data analysis, data interpretation, or writing of the report.

## Declaration of competing interest

Christina Engebrethsen: Lecture honoraria: Novartis.

Mari Rasmussen: Lecture honoraria: Pfizer, Astra Zeneca.

Bjørnar Gilje: Advisory Role: Daiichi Sankyo, AstraZeneca, Novartis and Lilly Nordic.

Egil Blix: Consulting/Advisory Role: AstraZeneca, Daiichi Sankyo, Eli Lilly, Novartis, Pfizer, Pierre Fabre, Roche.

Jürgen Geisler: Advisory board meeting: Novartis, AstraZeneca, Pfizer, Lilly, MSD and Daiichi-Sankyo. Research Funding: Novartis.

Per Eystein Lønning: Lecture honoraria: Dagens Medisin and Illumina. Consulting honoraria: Laboratorios Farmaceuticos Rovi.

Hans Petter Eikesdal: Honoraria: Amgen, Astra Zeneca, Bristol-Myers Squibb, Dagens Medisin, HAI interactive AS, Novartis, Pfizer, Pierre Fabre, Roche. Consulting or Advisory Role: Aptitude Health, Daiichi Sankyo, Eli Lilly, Gilead, Medac, MSD, Novartis, Pfizer, Pierre Fabre, Roche. Research Funding: Astra Zeneca, Illumina, Novartis, Pfizer. Expert Testimony: Pfizer.

Stian Knappskog: Lecture honoraria: Novartis, Pfizer. Research funding (to institution): Pfizer, Illumina, Astra Zeneca. Travel, Accommodations, Expenses: Astra Zeneca, Pierre Fabre.

The other authors declare that they have no known competing financial interests or personal relationships that could have appeared to influence the work reported in this paper.
