# Supplementary material for: Neoadjuvant endocrine therapy with sequential palbociclib and chemotherapy based on Ki67 status in stage II-III breast cancer: An open-label, phase II study
Source: Breast. 2025 Dec 10;85:104672. doi: 10.1016/j.breast.2025.104672 (PMC12813210; doi:10.1016/j.breast.2025.104672)
Supplement: Multimedia component 1 [file mmc1.docx]

**Appendix A: Supplementary Information**

**Neoadjuvant endocrine therapy with sequential palbociclib and chemotherapy based on Ki67 status in stage II-III breast cancer: an open-label, phase II study**

Christina Engebrethsen, Synnøve Yndestad, Mari E. Rasmussen, Emiel A. M. Janssen, Bjørnar Gilje, Egil S. Blix, Helge Espelid, Steinar Lundgren, Jürgen Geisler, Laura Minsaas, Reidun Lillestøl, Hildegunn S. Aase, Turid Aas, Per E. Lønning, Hans P. Eikesdal and Stian Knappskog

**Content**

[Supplementary Figure 1 2](#_Toc214440247)

[Supplementary Figure 2 3](#_Toc214440248)

[Supplementary Table 1 4](#_Toc214440249)

[Supplementary methods 5](#_Toc214440250)

[Immunohistochemistry (IHC) 5](#_Toc214440251)

[DNA and RNA analyses 6](#_Toc214440252)

[References 9](#_Toc214440253)

**Supplementary Figure 1**

**Fig. S1** Trial design for the phase II neoadjuvant PETREMAC study.

Patients with breast cancers >40 mm (arms A-D) or >20 mm (arms E-H), and no distant metastasis (M0) were included and allocated to eight different treatment arms, based on estrogen receptor (ER), progesterone receptor (PGR), HER2 expression and *TP53* mutation status. ER, PGR and HER2 analyses were performed at each local site, whereas *TP53* mutation analysis was a centralized analysis at Haukeland University Hospital (Bergen, Norway). *^1^ Before protocol amendment^27^. *^2^ After protocol amendment^27^.

AI, aromatase inhibitor; biop., biopsy; CDK4/6i, CDK4/6 inhibitor; cont., continued; cyclophosph., cyclophosphamide; mut, mutation; PARPi, PARP inhibitor; PR, partial response; TNBC, triple negative breast cancer; wt, wild-type.

# **Supplementary Figure 2**


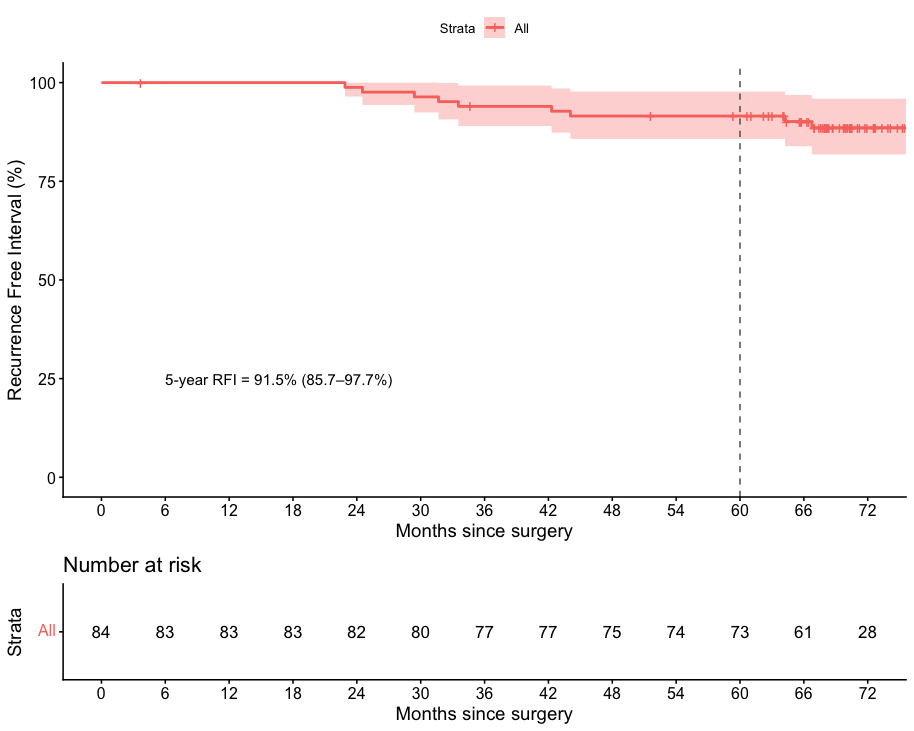


**Fig. S2** Relapse-free interval (RFI) for patients (n=84) in armA of the PETREMAC trial

At 5-years follow up (60 months; vertical dotted line), 7 patients had experienced relapse. (The RFI-curve also illustrates recorded follow-up beyond 60 months. Two additional patients have experienced relapse in the interval 60-70 month

# **Supplementary Table 1**

Adverse events registered for patients in arm A of the PETREMAC trial.

|  | **All grades (n)** | | **Grade 1-2 (n)** | | **Grade 3-4^1^ (n)** | |
| --- | --- | --- | --- | --- | --- | --- |
|  | NET +/- CDK4/6i | NAC | NET +/- CDK4/6i | NAC | NET +/- CDK4/6i | NAC |
| **Neutrophil count decreased** | 20 | 1 | 2 | 1 | 18 |  |
| **Peripheral sensory neuropathy** |  | 13 |  | 10 |  | 3 |
| **Gastrointestinal disorder** | 8 | 4 | 8 | 3 |  | 1 |
| **Skin disorders** |  | 9 |  | 7 |  | 2 |
| **Infection (any)** | 1 | 8 | 1 | 6 |  | 2 |
| **Fatigue/malaise** | 2 | 6 | 2 | 3 |  | 3 |
| **Fever** | 1 | 7 | 1 | 7 |  |  |
| **Increased liver enzymes** | 3 | 1 | 2 |  | 1 | 1 |
| **Edema limbs** |  | 4 |  | 3 |  | 1 |
| **Febrile neutropenia** |  | 3 |  | 2 |  | 1 |
| **Myalgia** | 1 | 2 | 1 | 1 |  | 1 |
| **Platelet count decreased** | 1 | 2 | 1 | 2 |  |  |
| **Anemia** |  | 2 |  | 2 |  |  |
| **Sinus tachycardia** |  | 2 |  | 2 |  |  |
| **Dyspnea** |  | 2 |  | 2 |  |  |
| **Watering eyes** |  | 2 |  | 2 |  |  |
| **Thromboembolic event** |  | 1 |  | 1 |  |  |
| **Urinary tract pain** | 1 |  | 1 |  |  |  |
| **Headache** | 1 |  | 1 |  |  |  |
| **Depression** | 1 |  | 1 |  |  |  |
| **Sum** | **40** | **69** | **21** | **54** | **19** | **15** |

Known side effects, listed in the SmPC for study drugs already approved, were not recorded as adverse events (AEs) unless a serious adverse event (SAE) occurred. AE reports noted after surgery or defined as «Not related/ not suspected related to study drug» are not listed. CTCAE v4.03 was used for AE scoring.

^1^Only two grade 4 events reported; decreased neutrophil count (n=1) and febrile neutropenia (n=1).

N, the number of times an event was reported, some patients had more than one AE reported; NET, neoadjuvant endocrine therapy; CDK4/6i, CDK4/6 inhibitor; NAC, neoadjuvant chemotherapy.

# **Supplementary methods**

## Immunohistochemistry (IHC)

Formalin-fixed paraffin-embedded tissue sections (2µm) were mounted on SuperFrost® Plus slides (Menzel Gläser, Braunschweig, Germany). Following incubation at 60°C for 1h, slides were transferred to the Dako Omnis (Dako, Glostrup, Denmark), and stained for Ki67 and E-cadherin using pre-optimized protocols. The Ki67 primary anti-body (clone MIB-1, DAKO, Glostrup, Denmark) was used at dilution 1:100 and visualized with the EnVision FLEX detection system (Dako). For E-cadherin, the primary antibody from Clone 36B5 (Novocastra, product code NCL-L-E-Cad) was used at dilution 1:50. Sections were counterstained with hematoxylin.

Ki67 scoring was a preplanned analysis performed prospectively during neoadjuvant treatment in the PETREMAC trial and was performed according to the Norwegian Breast Cancer Group guidelines (NBCG.no). At least 500 tumor cells were counted (at high magnification, 400x) in the area with the highest Ki67 positivity (selected at low magnification, 25x). All tumor cells were counted as either positive or negative in at least one field of vision (400x), where a percentage of positivity was scored by an experienced pathologist.

E-cadherin staining was evaluated using a scale of 0 to 3+, where 0 was no staining or <10% of the tumor cells were positive. A faint/barely perceptible membrane staining in over 10% of the tumor cells was 1+, and 2+ was a weak to moderate complete membrane staining in over 10% of the tumor cells, and 3+ was more than 30% of the tumor cells having strong complete staining. Staining for E-cadherin was performed retrospectively on all pretreatment samples except for two, where no tissue was available, to ensure that all ILC tumors were identified, and scoring was performed by two independent researchers. Where inconsistent scoring occurred, samples were reexamined jointly, a consensus decision was made, or a third party was consulted.

## DNA and RNA analyses

*DNA sequencing and mutation calling*

DNA extraction and library preparation were done using QIAamp DNA mini kit (Qiagen, Germany) and the SureSelect XT protocol (Agilent, US), previously described (1). Pretreatment breast cancer biopsies underwent targeted DNA sequencing of 360 cancer-related genes (2). Centralized DNA analysis was performed on an Illumina MiSeq sequencer at Haukeland University Hospital, Bergen, Norway, as described previously (1). The average sequencing depth was 250x and tumor mutation calling was assessed by CaVEman (3) and Pindel (4), with matched normal DNA from peripheral blood leucocytes (1).

A Variant Allele Frequency (VAF) cut-off of 0.05 was used, with a minimum absolute number of reads set at three, and a minimum sequencing depth, DP (total depth of coverage), of 30 to call a mutation. Identified genomic aberrations were filtered against normal leucocyte DNA. Mutations reported in Ensembl with frequency >1% in the population were defined as benign mutations. All called mutations were manually inspected in IGV for technical artefacts.

Driver mutations were defined by the following criteria: Driver mutations must be non-synonymous and have a minimum of four confirmed cases of the mutation reported in the COSMIC database (5). Regardless of previous COSMIC reports, frameshift mutations and nonsense mutations in tumor suppressor genes were called driver mutations. Apart from *TP53* mutation status which was used to allocate each patient to the correct arm of the PETREMAC trial, all other mutation calling was performed retrospectively.

*RNA isolation, library preparation and sequencing*

Total RNA was extracted from tumor tissue, using the RNeasy Mini Kit with on-column DNase digestion according to the manufacturer’s protocol (Qiagen, Valencia, CA). 10–30 mg of tumor tissue was used as input. RNA quality was assessed on a NanoDrop spectrophotometer and concentration was determined on a Qubit spectrophotometer. RNA-RIN values were estimated using the RNA 6000 Nano assay (Agilent Technologies, Santa Clara, CA) on a Bioanalyzer 2100. Minimum RIN-value for further analyses was of 7, except for cases where no replacement material was available, here RIN >6 was accepted.

According to the manufacturer’s protocol, 300-500 ng total RNA input was used with the TruSeq Stranded Human Total RNA Ribozero Gold Library Prep Kit (Illumina, San Diego, CA). Average sizes and quality of the resulting libraries were assessed using the DNA 1000 assay (Agilent Technologies, Santa Clara, CA) on a Bioanalyzer 2100. Quantification of libraries was performed by real-time PCR using KAPA Library Quantification kits (Kapa Biosystems, Wilmington, MA) on a Lightcycler 480 II instrument (Roche). Libraries were normalized to a pooled library concentration of 2,05-2,15 nM and sequenced on a NovaSeq 6000 (Illumina) using 2x100 cycles. This provided a yield of minimum of 70 million reads per sample.

BCL files were processed using the DRAGEN^TM^ Bio-IT Platform v3.8 (Illumina). Samples were demultiplexed using the DRAGEN BCL converter. Gene expression data were processed using the DRAGEN RNA pipeline, with mapping against GRCh38. Duplicate markings were enabled but not removed. The annotation file (.gtf) for ALT-aware GRCh38 from GENCODE (6) was used.

*Gene expression profiling and risk scores*

A post hoc gene expression analysis was performed based on RNA sequencing to assign the tumors to PAM50 breast cancer subgroups. Genes with 0 counts were removed and raw expression data were normalized by VST transformation using DESeq2(7). Then the PAM50 subtypes were assigned with the R package Genefu (8) using the centroids published by Parker et al (9).

EndoPredict (10) (including information on nodal status), Gene70/ MammaPrint (11), and Oncotype DX (12) risk scores were calculated as published using Genefu with the provided official cutoff values for stratification for high/low-risk score. In brief, tumors with EndoPredict score above 3.3 are defined as high-risk (10), while the Gene70/ Mammaprint cutoff is -0.3. Oncotype DX cutoff points: low risk < 18), intermediate risk ≥18 but <31, and high risk ≥ 31. PAM 50 ROR-C (9) was calculated using a modified Genefu function (rorS) to calculate ROR-C including tumor size, where the weights were adjusted according to Parker 2009 (ROR-C = 0.05 • basal + 0.11 • HER2 - 0.23 • LumA + 0.09 • LumB + 0.17 • (Tumor size)). Risk stratification to high/intermediate/low-risk combines the use of nodal status and ROR-C score as published in the ABCSG-8 trial (13): Node-negative 0-40 = Low, 41-60 = Intermediate, 61-100 = High; Node-positive (1-3 nodes) 0-15 = Low, 16-40 = Intermediate, 41-100 = High; for Node-positive (≥ 4 nodes) 0-100 = High.

# **References**

1. Eikesdal HP, Yndestad S, Elzawahry A, Llop-Guevara A, Gilje B, Blix ES, et al. Olaparib monotherapy as primary treatment in unselected triple negative breast cancer. Annals of Oncology. 2021;32(2):240-9.

2. Yates LR, Gerstung M, Knappskog S, Desmedt C, Gundem G, Van Loo P, et al. Subclonal diversification of primary breast cancer revealed by multiregion sequencing. Nature medicine. 2015;21(7):751-9.

3. Jones D, Raine KM, Davies H, Tarpey PS, Butler AP, Teague JW, et al. cgpCaVEManWrapper: Simple Execution of CaVEMan in Order to Detect Somatic Single Nucleotide Variants in NGS Data. Current Protocols in Bioinformatics. 2016;56(1):15.0.1-.0.8.

4. Raine KM, Hinton J, Butler AP, Teague JW, Davies H, Tarpey P, et al. cgpPindel: Identifying Somatically Acquired Insertion and Deletion Events from Paired End Sequencing. Current Protocols in Bioinformatics. 2015;52(1):15.7.1-.7.2.

5. Forbes SA, Beare D, Boutselakis H, Bamford S, Bindal N, Tate J, et al. COSMIC: somatic cancer genetics at high-resolution. Nucleic Acids Res. 2017;45(D1):D777-d83.

6. GENCODE. Human, Release 46 (GRCh38.p14). 2024.

7. Love MI, Huber W, Anders S. Moderated estimation of fold change and dispersion for RNA-seq data with DESeq2. Genome Biology. 2014;15(12):550.

8. Deena M.A. Gendoo NR, Markus S. Schroeder, Laia Pare, Joel S Parker, Aleix Prat, Benjamin Haibe-Kains. genefu: Computation of Gene Expression-Based Signatures in Breast Cancer 2022 [R package version 2.28.0:[Available from: <http://www.pmgenomics.ca/bhklab/software/genefu>.

9. Parker JS, Mullins M, Cheang MC, Leung S, Voduc D, Vickery T, et al. Supervised risk predictor of breast cancer based on intrinsic subtypes. Journal of clinical oncology : official journal of the American Society of Clinical Oncology. 2009;27(8):1160-7.

10. Filipits M, Rudas M, Jakesz R, Dubsky P, Fitzal F, Singer CF, et al. A New Molecular Predictor of Distant Recurrence in ER-Positive, HER2-Negative Breast Cancer Adds Independent Information to Conventional Clinical Risk Factors. Clinical Cancer Research. 2011;17(18):6012-20.

11. van 't Veer LJ, Dai H, van de Vijver MJ, He YD, Hart AAM, Mao M, et al. Gene expression profiling predicts clinical outcome of breast cancer. Nature. 2002;415(6871):530-6.

12. Paik S, Shak S, Tang G, Kim C, Baker J, Cronin M, et al. A Multigene Assay to Predict Recurrence of Tamoxifen-Treated, Node-Negative Breast Cancer. New England Journal of Medicine. 2004;351(27):2817-26.

13. Gnant M, Filipits M, Greil R, Stoeger H, Rudas M, Bago-Horvath Z, et al. Predicting distant recurrence in receptor-positive breast cancer patients with limited clinicopathological risk: using the PAM50 Risk of Recurrence score in 1478 postmenopausal patients of the ABCSG-8 trial treated with adjuvant endocrine therapy alone. Annals of Oncology. 2014;25(2):339-45.
